# Dipeptide alanine-glutamine ameliorates retinal neurodegeneration in an STZ-induced rat model

**DOI:** 10.3389/fphar.2024.1490443

**Published:** 2024-11-19

**Authors:** Yuhan Zhang, Mingyan Wei, Xin Wang, Yuan Xu, Rongrong Zong, Xiang Lin, Shiying Li, Wensheng Chen, Zuguo Liu, Qian Chen

**Affiliations:** ^1^ Xiamen University Affiliated Xiamen Eye Center, Fujian Provincial Key Laboratory of Ophthalmology and Visual Science, Fujian Engineering and Research Center of Eye Regenerative Medicine, Eye Institute of Xiamen University, School of Medicine, Xiamen University, Xiamen, China; ^2^ Department of Ophthalmology, Xiang’an Hospital of Xiamen University, Xiamen, Fujian, China; ^3^ Department of Ophthalmology, The First Affiliated Hospital of Xiamen University, Xiamen, Fujian, China

**Keywords:** diabetic retinopathy, alanine-glutamine, retinal neurodegeneration, glucose metabolism, mTOR

## Abstract

**Introduction:**

Diabetic retinopathy (DR) is a common complication of diabetes. Retinal neuronal degeneration is an early event in DR, indicated by the declined electroretinogram (ERG). Dipeptide alanine-glutamine (Ala-Gln) is widely used as a nutritional supplement in the clinic and has anti-inflammatory effects on the gastrointestinal system. Studies also reported that glutamine has beneficial effects on diabetes. This study aimed to investigate the possible therapeutic effects of Ala-Gln in diabetic retinal neurodegeneration and to delineate its mechanism of action.

**Methods:**

The Streptozotocin (STZ)-induced rat model was used as a DR model. ERG was used to measure the neuronal function of the retina. Western blot analysis was performed to test the expression of proteins. Immunofluorescence staining was used for the detection and localization of proteins.

**Results:**

In diabetic rats, the amplitudes of ERG were declined, while Ala-Gln restored the declined ERG. Retinal levels of inflammatory factors were significantly decreased in Ala-Gln-treated diabetic rats. Ala-Gln mitigated the declined levels of glutamine synthetase and ameliorated the upregulated levels of glial fibrillary acidic protein (GFAP) in diabetic retinas. Moreover, Ala-Gln upregulated the glycolytic enzymes pyruvate kinase isozymes 2 (PKM2), lactate dehydrogenase A (LDHA) and LDHB and stimulated the mTOR signaling pathway in diabetic retinas. The mitochondrial function was improved after the treatment of Ala-Gln in diabetic retinas.

**Discussion:**

Ala-Gln ameliorates retinal neurodegeneration by reducing inflammation and enhancing glucose metabolism and mitochondrial function in DR. Therefore, manipulation of metabolism by Ala-Gln may be a novel therapeutic avenue for retinal neurodegeneration in DR.

## Introduction

Diabetic retinopathy (DR), a neurovascular complication of diabetes mellitus, has been the primary cause of blindness among the working-age population in Western countries (Cheung et al., 2010). In recent years, researchers have found that early neuronal dysfunction and neurodegeneration caused by DR may precede vascular pathology (Zhou et al., 2023). The contributions of microvascular and neural elements, the so-called neurovascular unit, to the pathophysiology of DR were recognized and emphasized (Simó et al., 2018). Dysregulation of metabolism has been shown to play a pathogenic role in DR. For example, studies have shown that diabetic retinas have dysregulation of glucose, amino acids, and lipid metabolism, which contributes to the development and/or progression of DR (Busik, 2021; Chen et al., 2022). The current treatment strategies for DR are limited, especially those for retinal neurodegeneration. Thus, manipulating the metabolism in diabetic retinas may have therapeutic effects on DR.

Glutamine is a conditionally essential amino acid and the most abundant in the blood. It serves as an essential nutrient for providing the primary energy supply, and a substitute for protein synthesis (Watford, 2015). When glutamine enters the cells via transporters, it can be metabolized to glutamate and enter the tricarboxylic acid (TCA) cycle for ATP production. The conversion of glutamine to glutamate is facilitated by mitochondrial glutaminases (GLS). The glutamate is reported to have toxic effects on the inner retinal layer and retinal ganglion cells (Lucas and Newhouse, 1957). However, the supplementation of glutamine by intraperitoneal injection or intravitreal injection showed no toxic effects on retinal cells (Lucas and Newhouse, 1957). The role of glutamine in tumor metabolism has been extensively studied (Altman et al., 2016). Cancer cells can rapidly use glutamine for energy generation and biomass accumulation (Windmueller and Spaeth, 1974). In addition, many studies have focused on the glutamate/glutamine cycle and glutamine anaplerosis (Lieth et al., 2000a; Bui et al., 2009; Goswami et al., 2024), but few studies have examined the effects of glutamine on retinal neurodegeneration.

Studies have shown that glutamine and glutamic acid were the most distinctive metabolites present in the plasma of patients with DR (13). Patients with proliferative diabetic retinopathy or proliferative vitreoretinopathy have significantly lower levels of glutamine in the vitreous humor compared to those with idiopathic preretinal macular fibrosis (Ishikawa et al., 1995). In addition, Rhee SY et al. reported the glutamine was decreased, while glutamic acid was increased in the plasma of the patients with DR (Rhee et al., 2018). Recently, a systematic review of metabolomics in DR showed that L-glutamine was a potential biomarker for DR (Hou et al., 2021). However, whether glutamine plays a protective or pathogenic role in the diabetic retinas is still unknown.

Ala-Gln, a dipeptide, is highly soluble in water. Ala-Gln has been used as an enteric energy supplement in the clinic (Vychytil et al., 2018). It was reported that Ala-Gln could reduce intestinal inflammation and protect the intestinal blood barrier (Badole et al., 2013). Total parenteral nutrition supplemented with Ala-Gln reduced infectious complications and stimulated insulin release in critically ill patients (Grau et al., 2011). Supplementation of glutamine in STZ-induced diabetic rats increased plasma levels of insulin and mitigated pancreatic islet apoptosis (Medras et al., 2018). Meanwhile, dipeptide Arginine-glutamine could inhibit neovascularization in oxygen-induced retinopathy (Neu et al., 2006), indicating that amino acids may be involved in retinal metabolism. In this study, we hypothesized that Ala-Gln might have beneficial effects on DR. Based on an STZ-induced diabetic rat model, we evaluated the effect of Ala-Gln on the retinal neuronal function. Meanwhile, its mechanism of action was also investigated. Our study demonstrated that supplementation of Ala-Gln ameliorates retinal neurodegeneration by reducing inflammation and enhancing glucose metabolism and mitochondrial function in DR.

## Methods and materials

### Animals

Sprague-Dawley (SD) rats were purchased from Beijing Vital River Laboratory Animal Technology Co., Ltd (Beijing, China). The animals were housed in a specific pathogen-free facility and maintained in 12-hour light and 12-hour dark cycles. All procedures with animals in this study were performed in accordance with the ARVO “Statement for the Use of Animals in Ophthalmic and Vision Research”. The animal protocols (“XMULAC20190022” and “XMULAC20220168”) were approved by the Xiamen University Experimental Animal Ethics Committee.

### *In Vivo* experimental procedures

SD rats (male, 190∼210 g) were randomly divided into the normal group and the diabetic group. The rats were injected intraperitoneally with a single dose of streptozotocin (STZ, 50 mg/kg) or vehicle (Citrate buffer), respectively. Random blood glucose was tested using blood obtained from the tail tip after 1 week of STZ injection. Rats with blood glucose higher than 16.7 mmol/L were considered to be hyperglycemia. Blood glucose levels and body weights of the rats were monitored every month. Seventy-five days after the onset of diabetes, the rats were injected intraperitoneally with Ala-Gln (2 g/kg) or vehicle (saline solution) for 15 successive days. After the treatment, the rats were subjected to an electroretinogram (ERG) test or sacrificed by carbon dioxide, and the eyeballs/retinas were harvested for further experiments.

## Visual function measurements

Full-field ERGs were performed on SD rats to estimate the visual function. SD rats were subjected to dark adaptation and SD rats were anesthetized with sodium pentobarbital (40 mg/kg). The pupils were dilated with tropicamide phenylephrine eye drops (Santen, pharmaceutical Co., LTD., Shiga plant, Japan). ERGs were recorded using the Diagnosys Celeris system (Diagnosys LLC, Lowell, United States). The centers of both the corneas were attached with electrodes that served as both light guides and stimulators. Normal saline was used to hydrate the corneas. All the above processes were under dim red light to preserve the dark adaption. The a-wave and b-wave of 1.0 cd ·s/m^2^ of both eyes were recorded and analyzed.

### Retinal thickness measurements

Retinal thickness was measured by Optical coherence tomography (Beijing HealthOlight Technology Co., Ltd., Beijing, China). After 3 months of diabetes, the rats were anesthetized and pupils were dilated as described above. High-resolution images were obtained in OCT scanning according to the manufacturer’s instructions. The total thickness of the retina (from the retinal nerve fiber layer to the retinal pigmental epithelium, including both layers) was measured at 200 pixels length relative to the optic nerve head (ONH) at both sides using the ImageJ software. The measurements of both sides were averaged to produce a single thickness value for each retina according to a published study (Ferguson et al., 2013).

### Metabolites extraction for LC-MC detection

Each sample was accurately weighed and placed into an Eppendorf tube. Then, 1,000 μL of chilled extraction solution (prepared at −20°C, composed of acetonitrile-methanol-water in a 2:2:1 ratio, and including a mixture of isotope-labeled internal standards) was added. The mixture was briefly vortexed for 30 s, followed by homogenization at 40 Hz for 4 min and sonication for 5 min in an ice bath. This process of homogenization and sonication was repeated three times. Afterward, the samples were incubated at −40°C for 1 h and then centrifuged at 12,000 rpm and 4°C for 15 min. An aliquot of 80 μL from the clear supernatant was carefully transferred to an autosampler vial in preparation for UHPLC-MS/MS analysis.

### UHPLC-MRM-MS/MS analysis

The separation via UHPLC was conducted on an Agilent 1290 Infinity II series UHPLC System (Agilent Technologies), equipped with a Waters ACQUITY UPLC BEH Amide column (100 mm × 2.1 mm, 1.7 μm). The mobile phase A was 1% formic acid in water, and the mobile phase B was 1% formic acid in acetonitrile. The temperature of the column was maintained at 35°C, while the auto-sampler temperature was kept at 4°C with an injection volume of 1 μL. For assay development, an Agilent 6460 triple quadrupole mass spectrometer (Agilent Technologies) was utilized, coupled with an AJS electrospray ionization (AJS-ESI) interface. The typical ion source settings were as follows: capillary voltage = +4,000/−3500 V, Nozzle Voltage = +500/−500 V, gas (N2) temperature = 300°C, gas (N2) flow = 5 L/min, sheath gas (N2) temperature = 250°C, and sheath gas flow = 11 L/min, with a nebulizer = 45 psi The MRM parameters for each target analyte were optimized using flow injection analysis by injecting a standard solution of each analyte into the API source of the mass spectrometer. Several of the most sensitive transitions were used in the MRM scanning mode to optimize the collision energy for each Q1/Q3 pair. The Q1/Q3 pair with the highest sensitivity and selectivity in the optimized MRM transitions for each analyte was selected as the “quantifier” for quantitative monitoring. The other transitions served as “qualifiers” to verify the characterization of the target analyte. Agilent MassHunter Work Station Software (B.08.00, Agilent Technologies) was employed for MRM data acquisition and processing.

### Western blot analysis

Western blot analysis was performed as previously described (Murray et al., 2013). Briefly, the retina samples were sonicated and lysed in radioimmunoprecipitation assay (RIPA) buffer with protease and phosphatase inhibitor cocktail. The protein concentrations were determined by bicinchoninic acid (BCA) assay. Equal amounts of protein (20 ug) were resolved by SDS–polyacrylamide gel electrophoresis and then transferred to a polyvinylidene fluoride (PVDF) film. The membranes were blocked and incubated with primary antibodies overnight at 4°C. After several washes, the membranes were incubated with secondary antibodies for 1 h at room temperature (RT). The blot signals were developed with an enhanced chemiluminescence reagent kit (NCM Biotech, Newport, RI, United States). The bands were quantified with an ImageJ density analyzer and normalized to β-actin levels. The antibodies were used for Western blot analysis in this study are shown in Table 1.

**TABLE 1:** Antibodies for western blot.

Primary antibody name	Brand	Catalog no.	Dilution
Glial fibrillary acidic protein (GFAP)	Abcam	ab7260	1: 1,000
Vascular endothelial growth factor (VEGF)	Santa Cruz	SC-7269	1: 1,000
Vascular cell adhesion molecule-1(VCAM-1)	Santa Cruz	SC-13160	1: 1,000
Intracellular adhesion molecule-1 (ICAM-1)	Santa Cruz	SC-8439	1: 1,000
Lactate dehydrogenase A (LDHA)	Proteintech	19987-1-AP	1: 2000
Lactate dehydrogenase B (LDHB)	Proteintech	14824-1-AP	1: 5,000
Phosphorylated- mammalian target of rapamycin (p-mTOR)	Cell Signaling Technology	5,536	1:1,000
Mammalian target of rapamycin (mTOR)	Cell Signaling Technology	2,983	1:1,000
Glutamine Synthetase (GS)	Sigma-Aldrich	MAB302	1: 1,000
Anti-β-actin	Sigma-Aldrich	A3854	1: 5,000

### Immunofluorescence staining

The preparation of embedded paraffin blots was performed as previously documented (Pang et al., 2021). Briefly, eyeballs are enucleated and fixed in 4% paraformaldehyde solution (PFA) for 24 h at RT. The PFA was removed and the eyeballs were rinsed with 1x phosphate-buffered saline (PBS) for 3 × 10 min. After a series of dehydration, the eyeballs were embedded in paraffin. The paraffin-embedded sections of retinas at the thickness of 6 μm were placed in a 60°C oven for 1 h and deparaffinized in xylene and then dehydrated in degraded ethanol. Sections were placed in 1x Tris/EDTA buffer and boiled for 10 min for antigen retrieval. After cooling down, sections were incubated with 0.5% Triton X-100 for 20 min, and blocked with 10% Bovine serum albumin (BSA) in 1X PBS for 60 min. Then, sections were separately incubated with primary antibodies overnight at 4°C. After washing three times with 1X PBS, the sections were incubated with Alexa Fluor 594-conjugated IgG (Abcam, Cambridge, United Kingdom) or Alexa Fluor 488-conjugated IgG (Abcam) for 60 min at RT. The nucleus was counterstained with 4′, 6-diabmidino-2-phenylindole (DAPI). The sections were covered with coverslips after adding an antifade mounting medium (Vector Laboratories, Newark, United States). Images were acquired using a Zeiss microscope system (LSM 880, Wetzlar, Germany). The images of immunostaining of individual proteins in the different groups were acquired using the same exposure time. The antibodies were used for immunostaining in this study are shown in Table 2.

**TABLE 2:** Antibodies for immunofluorescence.

Antibody name	Brand	Catalog no.	Dilution
Translocase of the inner mitochondrial membrane 23 (TIM23)	Proteintech	11123-1-AP	1: 500
Translocase of the outer mitochondrial Membrane 20 (TOM20)	Proteintech	11802-1-AP	1: 300
Pyruvate kinase M2 (PKM2)	Cell Signaling Technology	4,053	1: 200
Glial fibrillary acidic protein (GFAP)	Cell Signaling Technology	12,389	1:200
Glutamine Synthetase (GS)	Sigma-Aldrich	MAB302	1: 300
Alexa Fluor 594-conjugated goat anti-rabbit	Abcam	ab150080	1: 300
Alexa Fluor 488-conjugated goat anti-rabbit	Abcam	ab150077	1: 300
Alexa Fluor 594-conjugated goat anti-rat	Abcam	ab150160	1: 300
Alexa Fluor 488-conjugated goat anti-rat	Abcam	ab150157	1: 300

### Statistical methods

All the statistical data were analyzed by GraphPad Prism (GraphPad Software, Inc.) and presented as Means ± SEM. Comparisons between the two groups were performed using Student's *t*-test. Analysis of variance (ANOVA) was used when comparing two groups or more. A *p*-value of less than 0.05 was considered a statistically significant difference.

## Results

### Ala-Gln improves retinal neural function in diabetic rats

**FIGURE 1:**
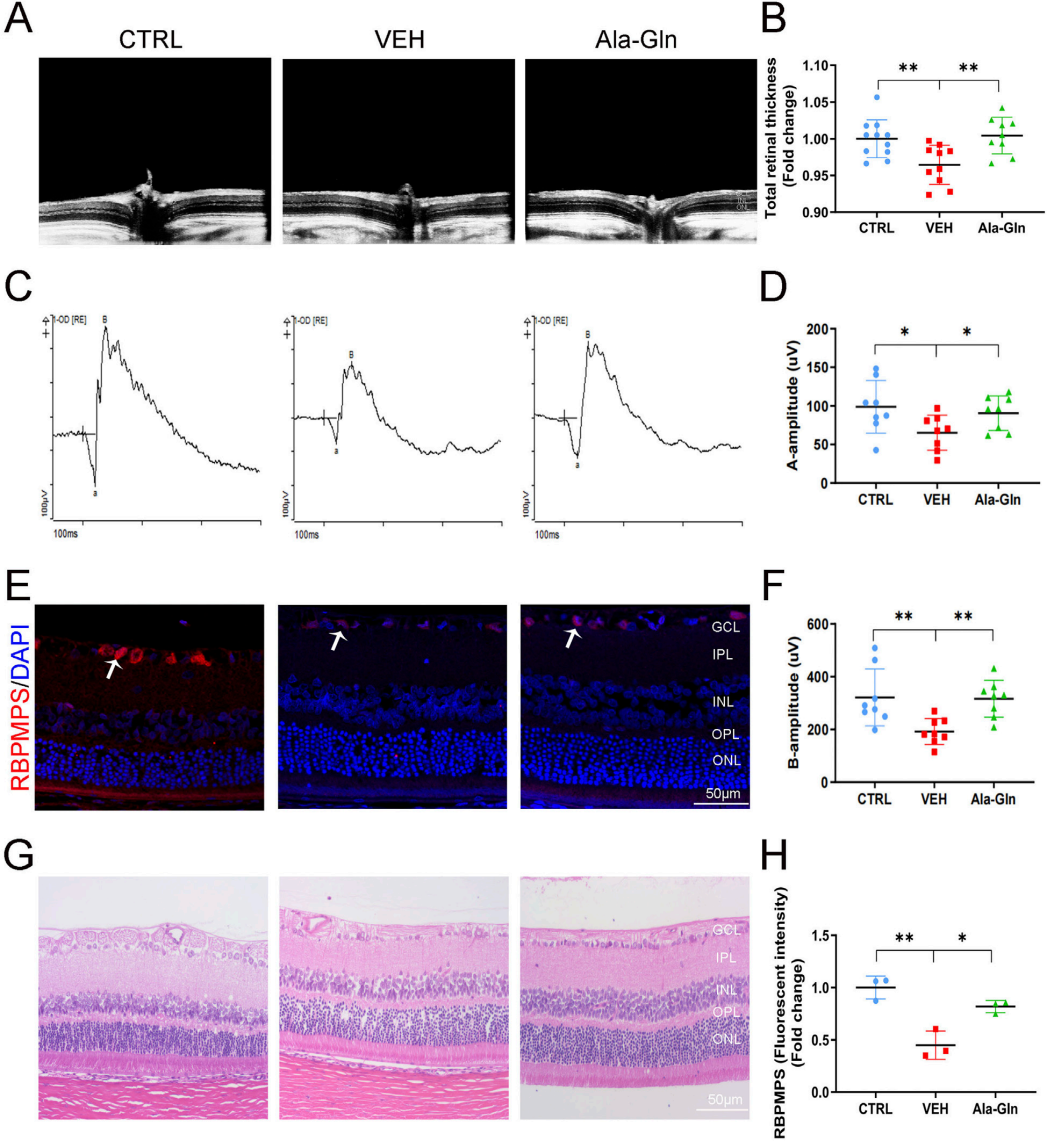
Ala-Gln improves retinal neural function in diabetic rats. **(A)** Retinal structure *in vivo* of the control group, VEH group, and Ala-Gln group was assessed by OCT. **(B)** Retinal thickness was assessed and quantified according to the OCT images from **(A)**. **(C)** ERG was obtained by averaging three responses to 1.0 cd s/m^2^ flashes **(D, F)** The amplitudes of ERG a-wave **(D)** and b-wave **(F)** of the three groups were analyzed and quantified. **(E)** Immunostaining of ganglion cell marker RNA Binding Protein mRNA Processing Factor (RBPMPS) in the retinal sections of the three groups. White arrows point out the positive stained retinal ganglion cells (red). **(H)** The immunofluorescence intensities of RBPMPS of the three groups were quantified. **(G)** Representative images of HE staining of retinal sections of the indicated three groups were shown. Mean ± SEM, n equal to one animal, n = 3–8. *p < 0.05, **p < 0.01, ***p < 0.001.

In this study, we first investigated the effects of Ala-Gln on DR in an STZ-induced diabetic rat model. Compared to the control rats, the STZ-induced diabetic rats showed declined body weights (Supplementary Figure S1A) and higher glucose levels (Supplementary Figure S1B), suggesting the successful establishment of diabetes. OCT images of the retinal structure of the indicated three groups are shown in Figure 1A. The total retinal thickness based on the OCT images was declined in diabetic retinas but reversed by the treatment of Ala-Gln (Figure 1B). Both a wave and b wave in ERG were significantly declined in STZ-induced diabetic rats compared with those in control rats (Figures 1C, D, F), suggesting that neural degeneration was present in diabetic rats. Interestingly, both a wave and b wave were improved after the Ala-Gln treatment (Figures 1D, F), indicating the neuroprotective role of Ala-Gln. Meanwhile, immunofluorescence showed that the signals of the ganglion cell marker RBPMPS were decreased in diabetic retinas, and Ala-Gln ameliorates the decreased RBPMPS (Figures 1E, H). HE staining of the retinas showed the retinal structure of all three groups was well organized, indicating no toxic effects of Ala-Gln on the retina (Figure 1G). Taken together, these data indicated that Ala-Gln improves retinal neural function in diabetic rats.

### Ala-Gln decreases retinal pro-inflammatory factors in diabetic rats

**FIGURE 2:**
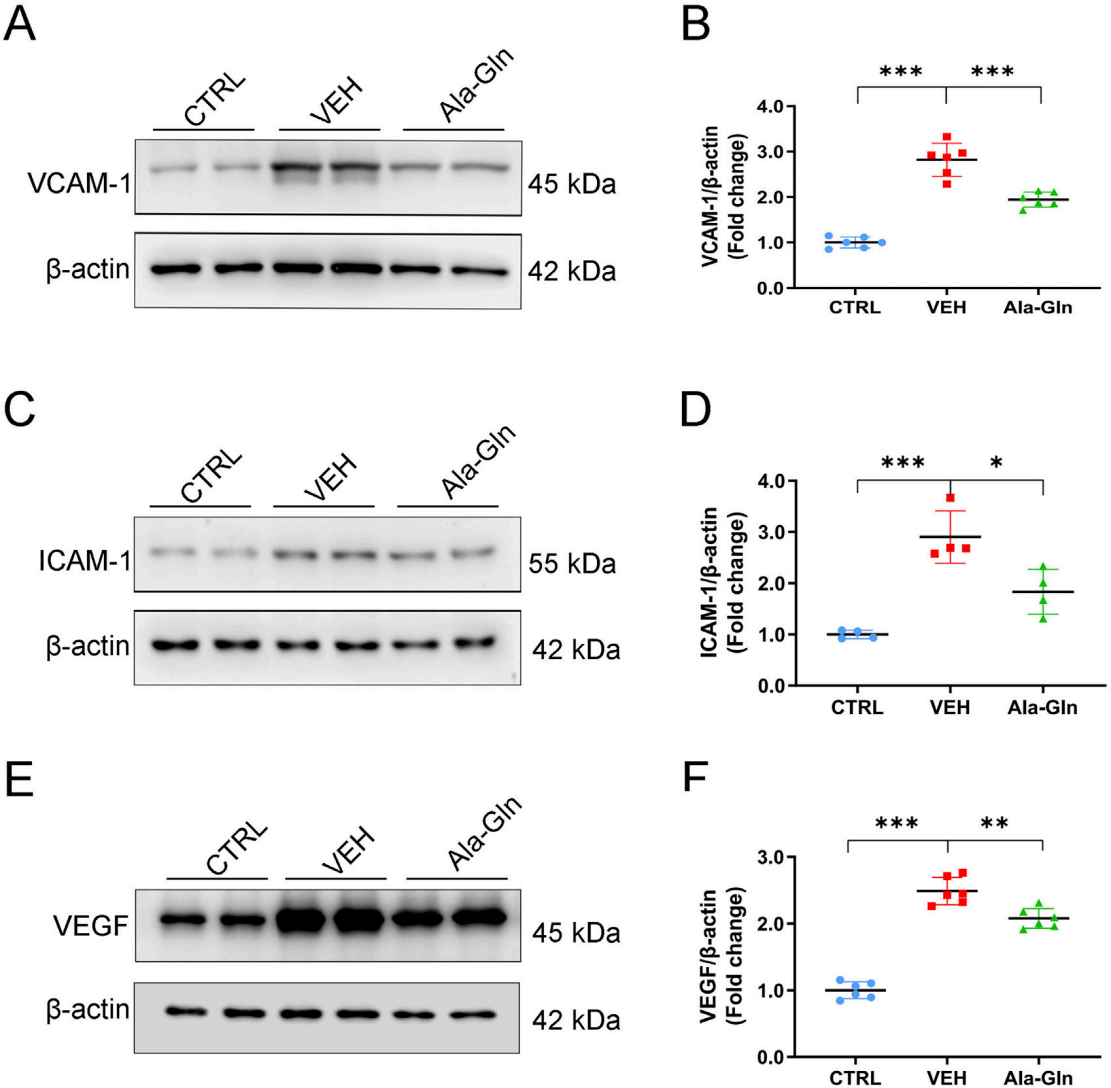
Ala-Gln reduces the levels of inflammatory cytokines in diabetic retinas. Representative blot images of VACM-1 **(A)**, ICAM-1 **(C)**, and VEGF **(E)** from the retinas of the control (non-diabetic) group, diabetic rats treated with vehicle (VEH), and diabetic rats treated with Ala-Gln. Retinal levels of VACM-1 **(B)**, ICAM-1 **(D)**, and VEGF **(F)** were quantified by densitometry relative to the actin levels. Mean ± SEM, n equal to one animal, n = 4–6, *p < 0.05, **p < 0.01, ***p < 0.001.

Next, we tested whether Ala-Gln has anti-inflammatory effects on diabetic retinas. Western blot analysis was performed to measure the protein expression of pro-inflammatory factors in the retinas of the control group, the vehicle group and the Ala-Gln group. Retinal levels of VCAM-1 (Figures 2A, B), ICAM-1 (Figures 2C, D), and VEGF (Figures 2E, F) were significantly increased in the vehicle group compared to those in the control group and were partially rescued in the Ala-Gln group, suggesting Ala-Gln have anti-inflammatory effects on diabetic retinas.

### Ala-Gln inhibits glial activation in diabetic retinas

**FIGURE 3:**
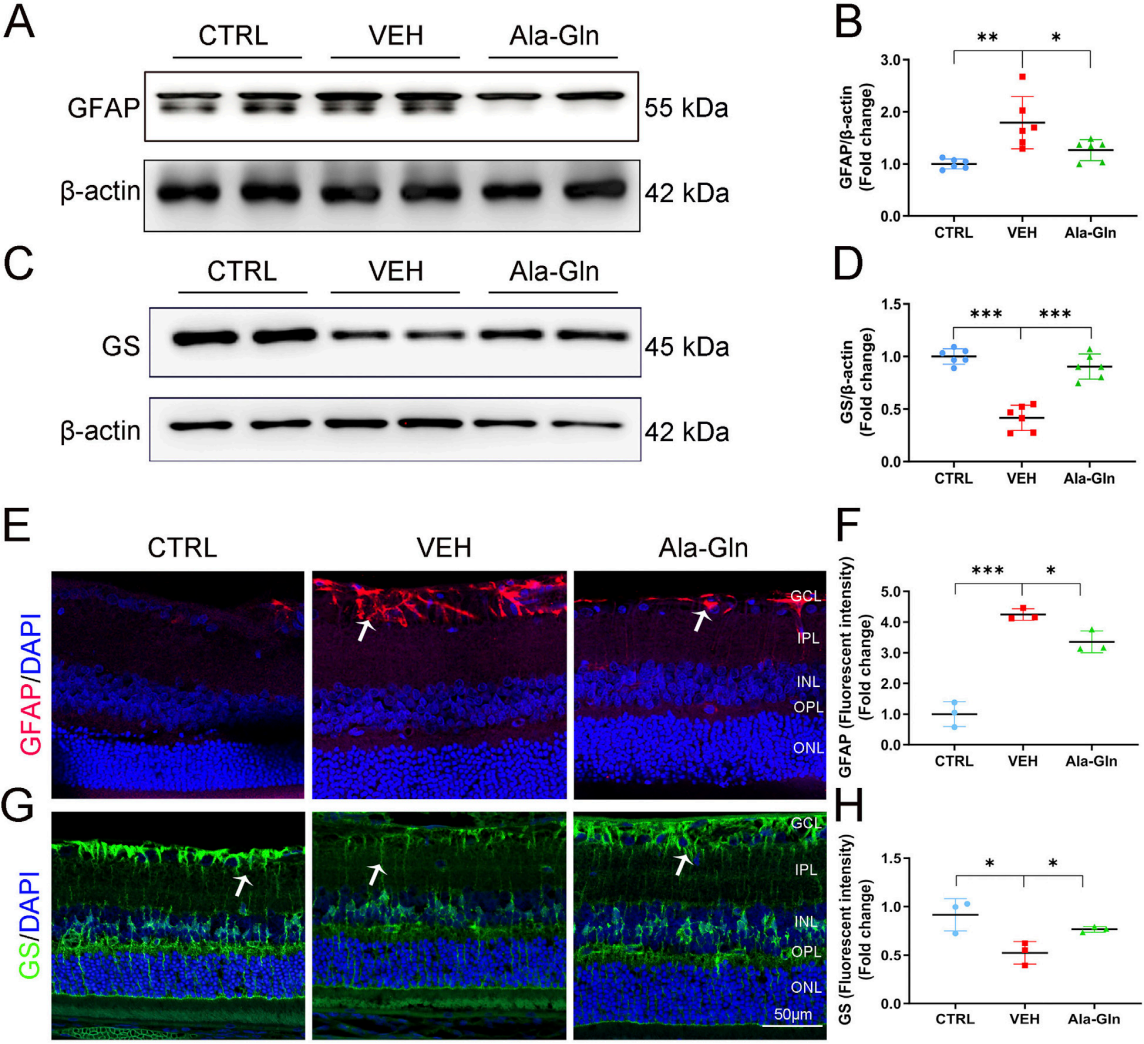
Ala-Gln ameliorates glial activation in the diabetic retina. The expression levels of GFAP **(A)** and GS **(C)** were detected by Western blot in the retinas of the control (non-diabetic) group, diabetic rats treated with vehicle (VEH) and diabetic rats treated with Ala-Gln. Levels of GFAP **(B)** and GS **(D)** were quantified by densitometry relative to the actin levels. Representative images of immunofluorescence staining of GFAP **(E)** and GS **(G)** in the retinal sections of the control group, the VEH group and the Ala-Gln group. White arrows indicate the GFAP-positive astrocytes in panel E (red). In panel G, white arrows point out the GS-positive Müller cells in the retina (green). The immunofluorescence intensities of GFAP **(F)** and GS **(H)** were quantified. Mean ± SEM; n equal to one animal, n = 3–6. *p < 0.05, **p < 0.01, ***p < 0.001.

Glial activation contributed to DR by increasing the secretion of pro-inflammatory factors and promoting retinal neovascularization (Zong et al., 2010; Gerhardinger et al., 2005). In this study, the protein levels of GFAP, a glial activation marker, were significantly increased in diabetic retinas (Figures 3A, B). Ala-Gln significantly reduced the expression of GFAP in diabetic retinas (Figures 3A, B), suggesting that Ala-Gln could suppress glial activity. Glutamine synthase (GS) catalyzes the transformation of glutamate into glutamine, which can detoxify the glutamate (Gorovits et al., 1997). The protein levels of GS were decreased in diabetic retinas, while levels of GS were significantly rescued in diabetic retinas after the treatment of Ala-Gln (Figures 3C, D). Consistently, the signal intensity of GFAP by immunostaining was much less in the retinal sections of the Ala-Gln group compared to those in the vehicle group (Figures 3E, F). The signal intensity of GS was decreased in the vehicle group and reversed by Ala-Gln (Figures 3G, H). Taken together, Ala-Gln increases the GS expression and decreases the GFAP expression, indicating a possibility of restoring the function of Müller cells and thus reducing the inflammation in the retina.

### Ala-Gln promotes glucose metabolism in diabetic retinas

**FIGURE 4:**
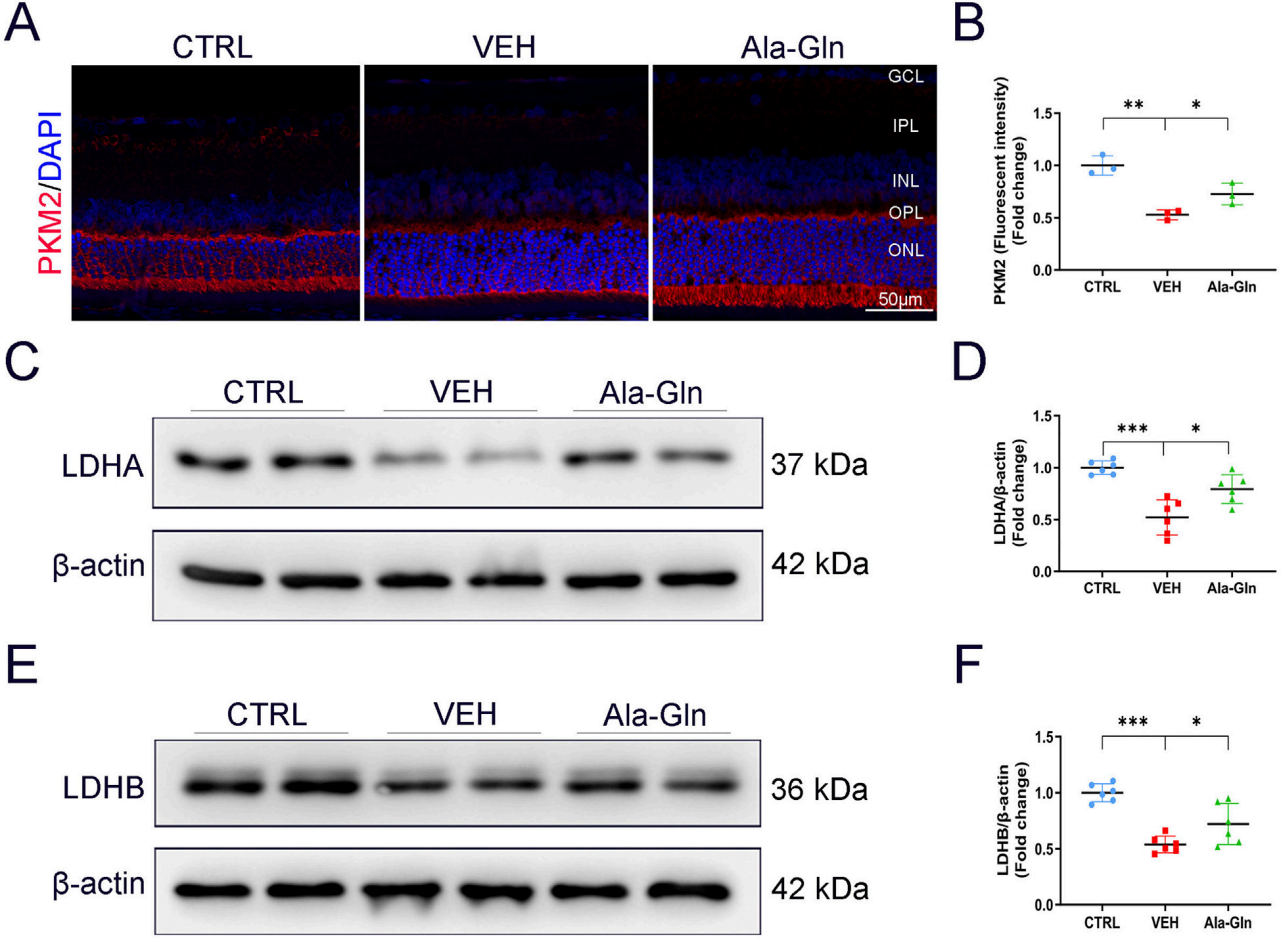
Ala-Gln stimulates retinal glycolysis in diabetic rats. **(A)** Representative images of immunofluorescence staining of PKM2 in the retinal sections of the control group, the VEH group, and the Ala-Gln group. **(B)** The immunofluorescence intensities of PKM2 in the three groups were quantified. The expression levels of LDHA **(C)** and LDHB **(E)** were detected by Western blot in the retinas of the three groups. Levels of LDHA **(D)** and LDHB **(F)** were quantified by densitometry relative to the actin levels. Mean ± SEM; n equal to one animal, n = 3–6. *p < 0.05, **p < 0.01, ***p < 0.001.

Since glutamine enters the TCA cycle for energy production, we investigated whether Ala-Gln could promote glucose metabolism in diabetic retinas. Results of immunostaining showed the signal intensity of PKM2, a critical enzyme in glucose metabolism, was slightly decreased in vehicle-treated diabetic retinas and was reversed/enhanced in the Ala-Gln treated diabetic retinas (Figures 4A, B). Lactate dehydrogenase (LDH) is the primary metabolic enzyme that converts pyruvate into lactate. The protein levels of LDHA (Figures 4C, D) and LDHB (Figures 4E, F) were significantly decreased in the diabetic retinas compared with those in the control retinas and were reversed after the treatment of Ala-Gln. Taken together, these results indicate that glycolysis is impaired in diabetic retinas, and Ala-Gln promotes glucose metabolism in diabetic retinas.

### Ala-Gln activates mTOR signaling and improves mitochondrial function in diabetic retinas

**FIGURE 5:**
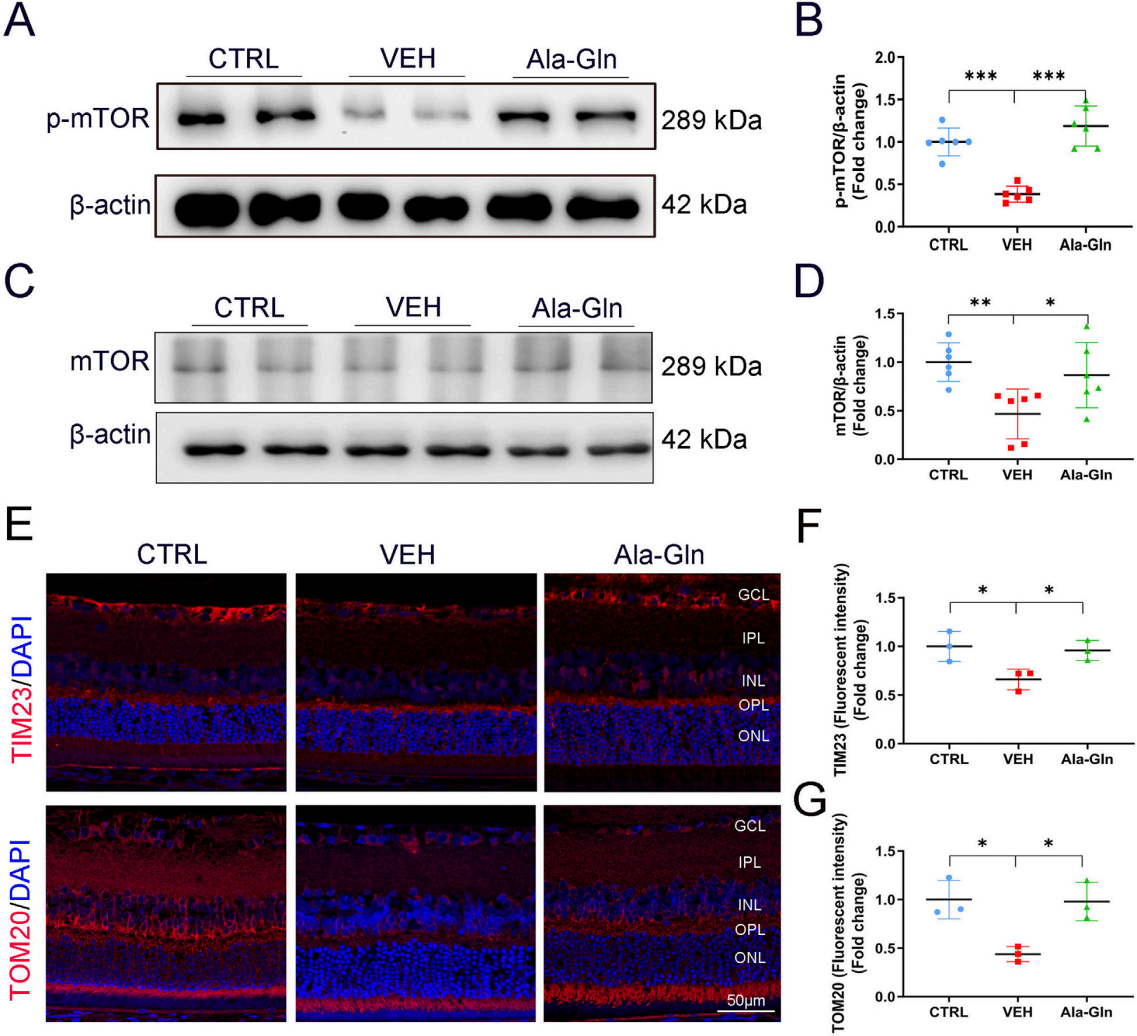
Ala-Gln activates mTOR signaling and improves mitochondrial function in diabetic retinas. The expression levels of p-mTOR **(A)** and mTOR **(C)** were detected by Western blot in the retinas of the control (non-diabetic) group, diabetic rats treated with vehicle (VEH) and diabetic rats treated with Ala-Gln. Protein levels of p-mTOR **(B)** and mTOR **(D)** were quantified by densitometry. **(E)** Representative images of immunofluorescence staining of TOM20 and TIM23 in the retinal sections of the three groups. The immunofluorescence intensities of TIM23 **(F)** and TOM20 **(G)** in the three groups were quantified. Mean ± SEM; n equal to one animal, n = 3–6. *p < 0.05, **p < 0.01, ***p < 0.001.

To explore the possible pathways that may promote glucose metabolism in the retina, the mTOR signaling pathway, a major signaling pathway that involves glucose metabolism, was measured. Both levels of p-mTOR and mTOR were decreased in diabetic retinas (Figures 5A–D). In contrast, Ala-Gln ameliorated the declined levels of p-mTOR and mTOR in diabetic retinas (Figures 5A–D). Further, immunostaining results showed the signal intensities of mitochondrial proteins TIM23 and TOM20 were decreased in diabetic retinas and restored by the treatment of Ala-Gln (Figures 5E–G). Taken together, these data suggest that Ala-Gln stimulates mTOR signaling and improves mitochondrial function in diabetic retinas.

## Supplementation of Ala-Gln does not increase the levels of glutamate in diabetic retinas

**FIGURE 6:**
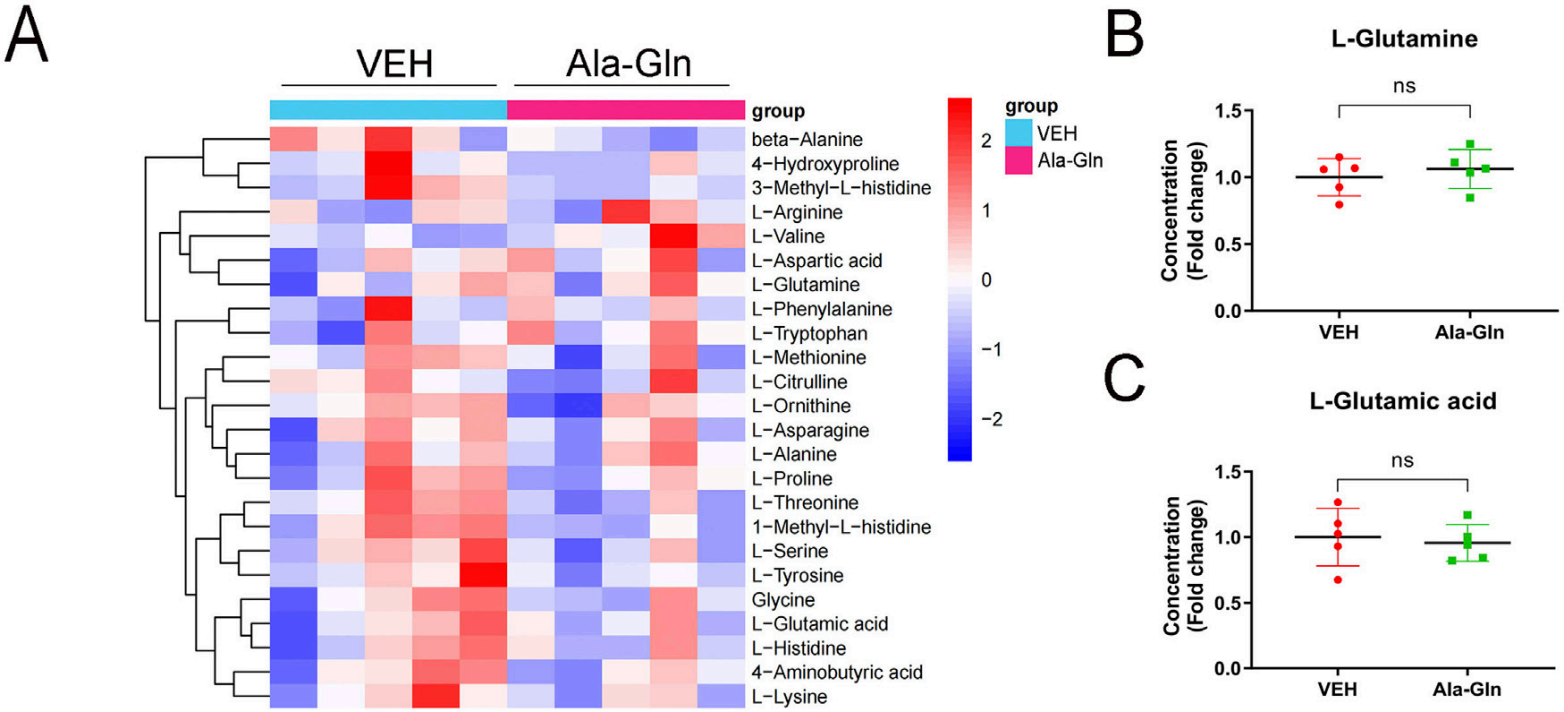
Levels of glutamate and glutamine in the retinas of diabetic rats treated with vehicle or Ala-Gln. Amino acid profile of the retinas of diabetic rats treated with vehicle (VEH) or Ala-Gln (Ala-Gln) using UHPLC-MRM-MS/MS. **(A)** Heat map of amino acid contents in the retina of the VEH group versus the Ala-Gln group **(B)** Levels of L-glutamine in the VEH group and Ala-Gln group. **(C)** Levels of L-glutamic acid in the VEH group and Ala-Gln group. Mean ± SEM; n equal to one animal, n = 5. Ns = non-significant.

To explore the levels of glutamine in diabetic rats, we performed amino acid sequencing to profile the amino acids in the control and diabetic retinas. All amino acids were listed and compared between the control and diabetic retinas (Supplementary Figure S2A). Among the changed amino acids, glutamate and glutamine are the most abundant amino acids in the retinas, and both glutamate and glutamine levels were significantly increased in diabetic retinas compared to those in controls (Supplementary Figures S2B and S2C). The amino acid profile of diabetic retinas treated with vehicle or Ala-Gln reveals all of the amino acids in the retinas (Figure 6A). Among them, the levels of L-glutamine and L-glutamic acid were not significantly changed in the diabetic retinas treated with vehicle or Ala-Gln (Figures 6B, C), suggesting that the extra supplementation of Ala-Gln in this study does not increase the retinal levels of glutamate in diabetic retinas.

## Discussion

Neuronal degeneration is an early event in DR, indicated by the decline of ERG. There is no specific treatment to prevent diabetic neuronal degeneration in DR. In this study, we have shown that supplementation of dipeptide Ala-Gln has anti-inflammatory and neuronal protective effects on DR. Ala-Gln suppresses glial activation and enhances glucose metabolism and mitochondrial function in the retina. Our study showed that supplementation of Ala-Gln has beneficial effects on DR, suggesting reprogramming metabolism by Ala-Gln may be a novel therapeutic avenue for retinal degeneration in DR.

Glutamine is conditionally essential, especially during stress conditions like injury, trauma, and infection, when organs require additional energy (Lacey and Wilmore, 1990). In this study, we have shown that Ala-Gln had anti-inflammatory effects in diabetic retinas by reducing the expression of inflammatory factors, which was consistent with other studies that showed anti-inflammatory effects of Ala-Gln (Grau et al., 2011; Ueno et al., 2011). For example, the supplementation of Ala-Gln reduced the infection rate of patients in intensive care units and improved the intestine barrier (Grau et al., 2011). Enteric Ala-Gln can enhance the function of immune systems and promote intestinal epithelial cell homeostasis (Ueno et al., 2011). Moreover, we have also found that Ala-Gln has neuronal protective effects in diabetic rats. Therefore, this protective effect may be due to reduced inflammation or enhanced glucose metabolism of photoreceptors and ganglion cells.

Müller cells are the most important glial cells in the retina, which span the whole retina and interact with many other types of retinal cells (Newman and Reichenbach, 1996). Müller cells play essential roles in maintaining the homeostasis of the retina. Glutamate is the major excitatory neurotransmitter in the retina. The glutamate is taken by glial cells and converted into non-toxic glutamine by GS in glial cells (Jafari-Vayghan et al., 2020). In diabetic retinas, the expression of GS is reduced, resulting in the accumulation of glutamate in the retina (Lieth et al., 2000b). Studies have shown that the altered glutamate levels in diabetic retinas are attributed to the dysfunction of Müller glial cell (Lieth et al., 1998). In this study, the protein levels of GS were decreased while GFAP was increased in diabetic retinas, which was consistent with other studies (Jafari-Vayghan et al., 2020; Gu et al., 2019; Chen et al., 2017). The supplementation of Ala-Gln decreased the GFAP levels, indicating the possible protective effects for glial cells. Specifically, the Ala-Gln increased GS levels, suggesting the function of glial cells may be rescued by Ala-Gln in diabetic retinas.

The isoform of pyruvate kinase PKM2 catalyzes phosphoenolpyruvate (PEP) to pyruvate in the process of glycolysis (Rajala, 2020). In this study, we have identified that levels of PKM2 were significantly reduced in STZ-induced diabetic retinas, consistent with a previous study that showed PKM2 was decreased in the retina of db/db mice, a type 2 diabetic animal model (Rajala et al., 2020). Supplementation of Ala-Gln rescued the expression of PKM2 levels in the diabetic retina, indicating that Ala-Gln may improve the glycolysis of cones and thus protect cones in diabetic rats. Lactate dehydrogenase is the primary metabolic enzyme converting pyruvate to lactate. Lactate is a fuel source for ATP/energy production through anaerobic glycolysis. About 80%∼90% of glucose used by photoreceptors was metabolized through anaerobic glycolysis (Petit et al., 2018). The proper lactate level in the retina plays a crucial role in preserving retinal ganglion cell function (Vohra et al., 2019). In this study, the decreased levels of LDHA and LDHB were ameliorated in diabetic retinas when the diabetic rats were treated with Ala-Gln, suggesting Ala-Gln enhanced glucose metabolism in diabetic retinas.

The mammalian target of rapamycin (mTOR) regulates cell proliferation, metabolism, autophagy, and apoptosis (Kaur and Sharma, 2017; Laplante and Sabatini, 2012). mTOR forms two distinct complexes, termed mTORC1 and mTORC2. mTOR is involved in many signaling pathways in the body, such as AMPK, PI3K/AKT, VAM6/Rag GTPases signaling (Laplante and Sabatini, 2012; Mao and Zhang, 2018). Dysfunction of mTOR signaling is associated with many diseases, including diabetes and neurological diseases (Kaur and Sharma, 2017). A study showed that mTORC1 stimulated glycolysis and glucose uptake by modulating the expression of HIF-1α (Düvel et al., 2010). Studies have also shown that mTORC1 stimulates glutamine metabolism and cell proliferation (Csibi et al., 2013; Csibi et al., 2014). Glutamine can also serve as a signaling molecule that activates Rag-mTORC1 signaling (Durán et al., 2012). In this study, the expression of mTOR was increased by Ala-Gln, suggesting that glutamine may stimulate the mTOR pathway to regulate glycolysis in diabetic retinas.

A controversy in this study is the toxicity of glutamate to the retina, which can be generated from glutamine via the tricarboxylic acid cycle. It is well established that glutamate is the major excitatory neurotransmitter involved in signal transmission in retinal neuronal cells. Multiple studies have shown that glutamate is excitotoxic for retinal photoreceptors and retinal ganglion cells (Lucas and Newhouse, 1957; Olney, 1982; Hansson, 1970). Meanwhile, studies have shown that levels of glutamate were increased in the retinas of diabetic animal models compared with those of the non-diabetic controls (Lieth et al., 2000b; Lieth et al., 1998), suggesting that elevated glutamate levels contributed to retina degeneration in diabetic animal models. In a previous study, the authors compared the effects of six groups of amino acids, including glutamate and glutamine, and identified the toxic effect of glutamate on the inner layer of retinas (Lucas and Newhouse, 1957). However, mice treated with glutamine showed no retinal abnormality, suggesting that glutamine is not toxic to the retina (Lucas and Newhouse, 1957). In contrast, glutamine was shown to have beneficial effects on patients with diabetes or diabetic animal models. Several studies have shown that the supplementation of glutamine resulted in a significant reduction in plasma glucose levels and increases in plasma and pancreatic insulin levels (Badole et al., 2013; Cruzat et al., 2015), suggesting the beneficial effects of glutamine in diabetes. Studies have also shown that supplementation of glutamine increased levels of glucagon-like peptide-1 (GLP-1) (Andersson et al., 2018; Tolhurst et al., 2011; Greenfield et al., 2009). In this study, supplementation of Ala-Gln did not significantly change the glutamate levels, indicating glutamine may have been metabolized through other pathways and was not directly converted to glutamate in diabetic retinas. Therefore, glutamine has beneficial effects instead of toxic effects in diabetic retinas. A future study is warranted to study glutamine metabolism in the retina, especially in diabetic retinas.

In summary, we have shown that Ala-Gln has protective effects on the neuronal function of diabetic retinas. Ala-Gln can reduce inflammatory cytokines and mitigate oxidative stress, probably by improving the synthesis of GS and decreasing GFAP expression. Ala-Gln enhances glucose metabolism and promotes mitochondrial function in diabetic retinas. Therefore, manipulating metabolism by Ala-Gln may serve as a therapeutic strategy in DR.

## Data Availability

The original contributions presented in the study are included in the article/Supplementary Material, further inquiries can be directed to the corresponding authors.
