# Supplementary material for: Dipeptide alanine-glutamine ameliorates retinal neurodegeneration in an STZ-induced rat model
Source: Front Pharmacol. 2024 Nov 19;15:1490443. doi: 10.3389/fphar.2024.1490443 (PMC11611560; doi:10.3389/fphar.2024.1490443)

***Supplementary Material***

**Part I Supplemental Figures and Figure Legends**

**Supplemental Figure 1. The successful establishment of diabetes in Sprague-Dawley rats.** (A) The body weights of diabetic rats were significantly reduced after the duration of diabetes for 1, 2, and 3 months, respectively. (B) The levels of blood glucose were significantly increased in the diabetic rats compared to the control rats after induction of diabetes for 1, 2, and 3 months, respectively. Mean ± SEM, n=8-10, **P<0.01，***P<0.001.


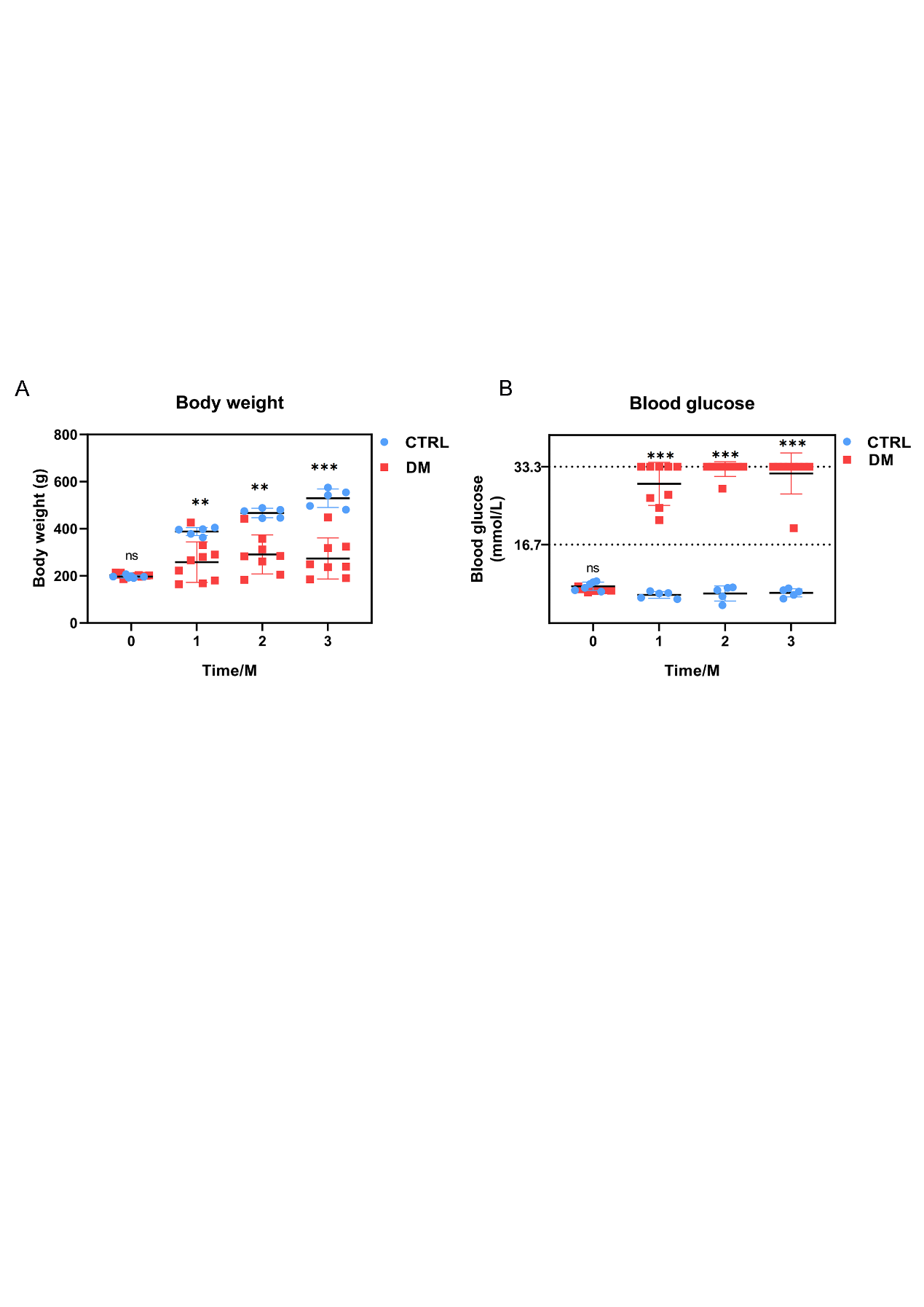


Supplemental figure 1

**Supplemental Figure 2. Levels of glutamine and glutamic acid in normal rat retinas and diabetic rat retinas.** After three months duration of diabetes, the retinas from the control group and the diabetic group were harvested. UHPLC-MRM-MS/MS analysis was used to detect the amino acids in the retinas of the control group (CTRL) and diabetic rat group (VEH), respectively. (A) Heat map of changes in amino acid contents in control VS VEH groups. (B) The levels of glutamine in the control and VEH group. (C) Levels of glutamic acid in the control and VEH groups. Mean ± SEM, n=8, *P<0.05, **P<0.01.

**
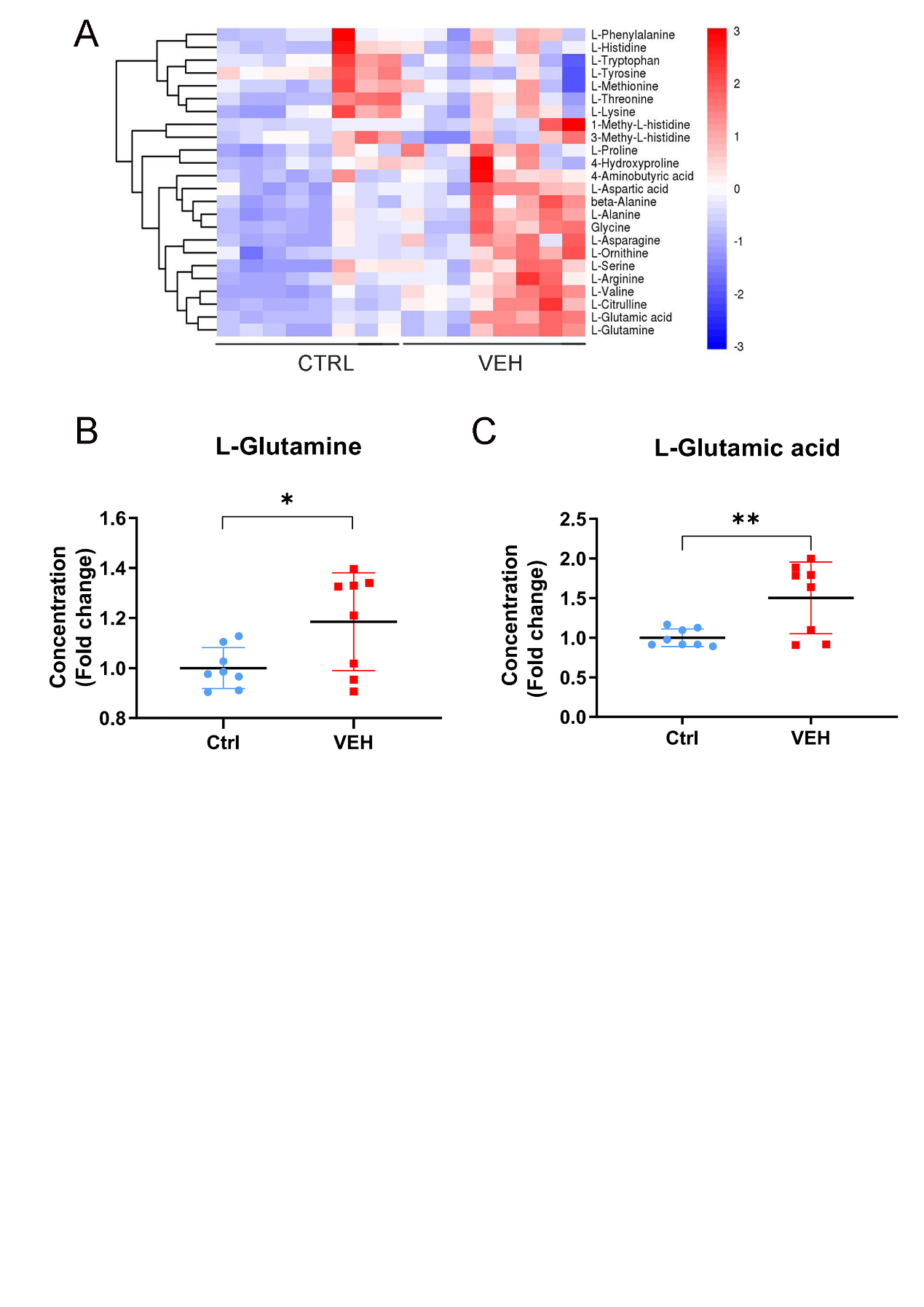
**

Supplemental figure 2

**Part II The original images of Western blot**.


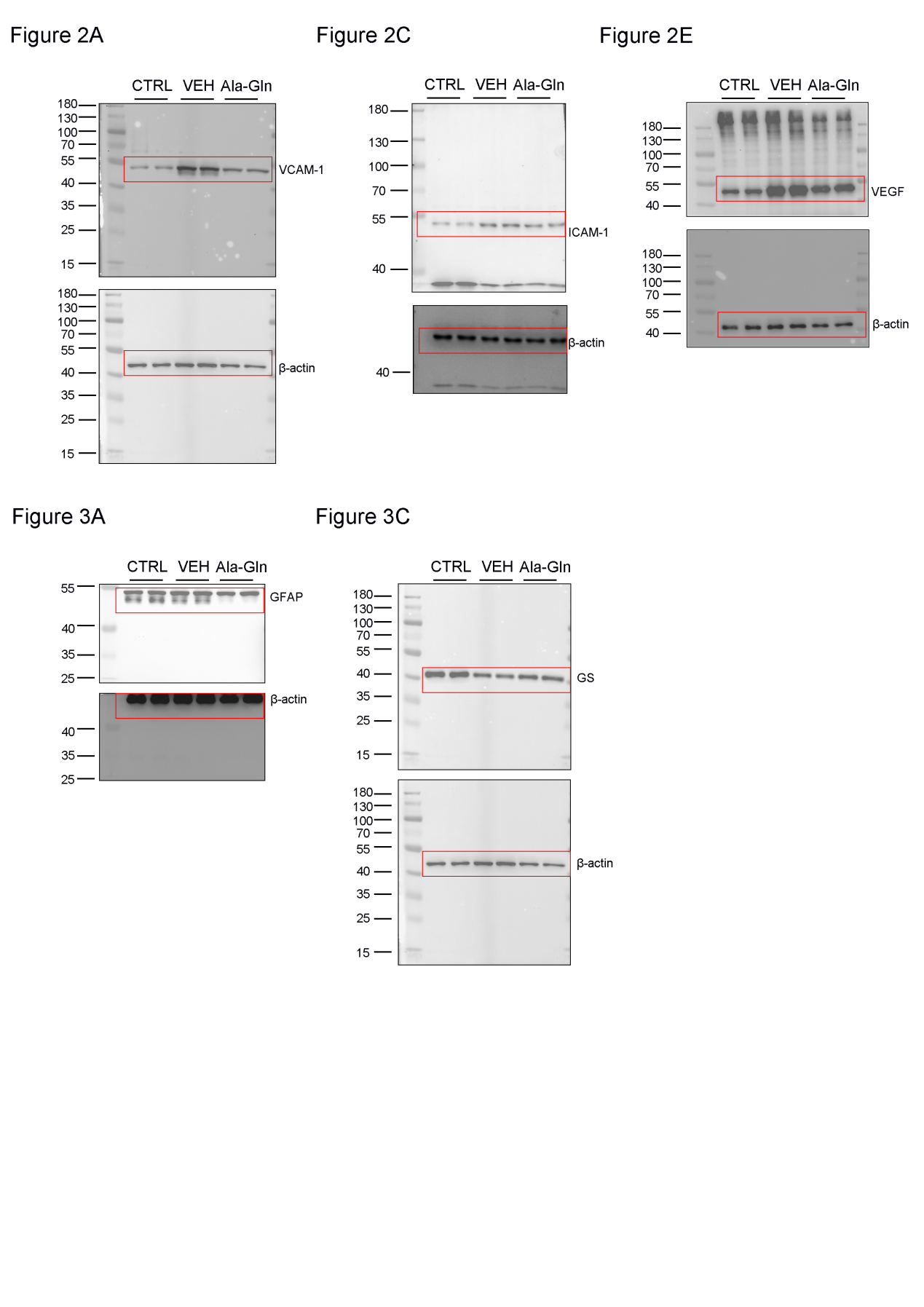

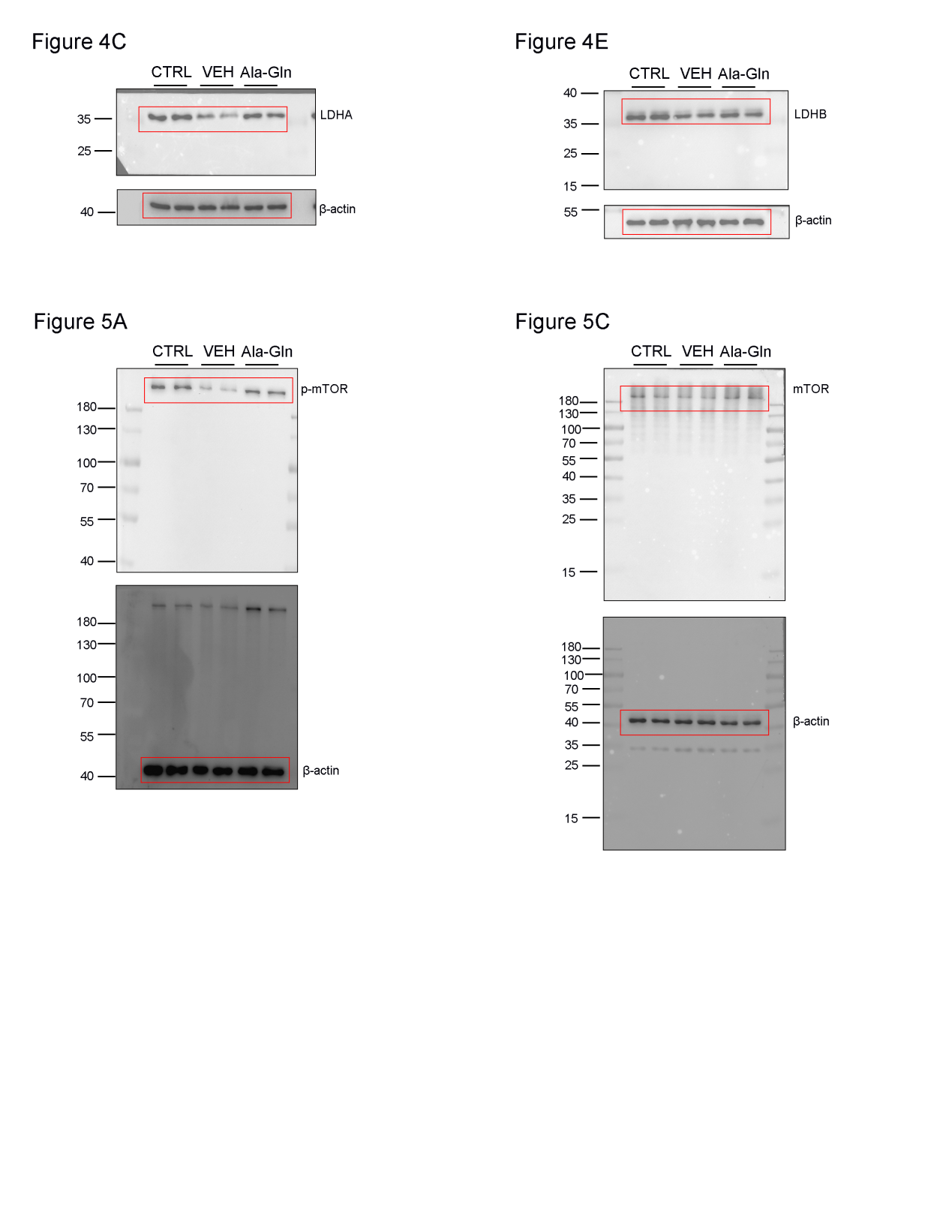

Supplement: Supplementary file 1 [file DataSheet1.docx]
